# Smartphones in ecology and evolution: a guide for the app-rehensive

**DOI:** 10.1002/ece3.888

**Published:** 2013-12-02

**Authors:** Amber G F Teacher, David J Griffiths, David J Hodgson, Richard Inger

**Affiliations:** 1 Environment and Sustainability Institute, University of ExeterPenryn Campus, Penryn, Cornwall, TR10 9EZ, U.K; 2 FoAM vzwKoolmijnenkaai 30–34, 1080, Brussels, Belgium; 3 Centre for Ecology and Conservation, Biosciences, College of Life and Environmental Sciences, University of Exeter, Penryn CampusPenryn, Cornwall, TR10 9EZ, U.K

**Keywords:** App, datalogger, georeference, global positioning systems, mobile, smartphone, technology.

## Abstract

Smartphones and their apps (application software) are now used by millions of people worldwide and represent a powerful combination of sensors, information transfer, and computing power that deserves better exploitation by ecological and evolutionary researchers. We outline the development process for research apps, provide contrasting case studies for two new research apps, and scan the research horizon to suggest how apps can contribute to the rapid collection, interpretation, and dissemination of data in ecology and evolutionary biology. We emphasize that the usefulness of an app relies heavily on the development process, recommend that app developers are engaged with the process at the earliest possible stage, and commend efforts to create open-source software scaffolds on which customized apps can be built by nonexperts. We conclude that smartphones and their apps could replace many traditional handheld sensors, calculators, and data storage devices in ecological and evolutionary research. We identify their potential use in the high-throughput collection, analysis, and storage of complex ecological information.

## Introduction

The rise of mobile technology continues apace and is beginning to provide remarkable opportunities for use in ecological and evolutionary research (e.g., see Snaddon et al. 2013). In essence, smartphones are portable, internet-enabled computers with a variety of sensors, providing us with a set of powerful research tools for collecting data. Smartphones provide the user with increased computational abilities, particularly internet access, global positioning systems (GPS), access to geographical information systems (GIS), microphones, accelerometers, and cameras with the capability not only to take high-resolution photographs, but also to read QR/barcodes and record video. These tools are accessible to an increasingly large number of people: By the end of 2012, there were approximately 4.4 billion mobile phone subscriptions globally, and around 15–20% of worldwide phone subscriptions are smartphones (Ericsson 2013). Subscriptions are also rising: Approximately 40% of mobile phones sold in 2012 were smartphones, up from 30% in 2011 (Ericsson 2013). This combination of computational power, sensors, and wide-scale user uptake means that apps provide an unprecedented opportunity for mass data collection from the public, while automating quality control and data management (Dufau et al. 2011; Graham et al. 2011; Newman et al. 2012). Indeed, smartphones have eloquently been described as the “butterfly nets of the 21st Century” by the developers at the New York start-up “Networked Organisms.”

Many existing apps serve as interfaces for citizen science data collection projects (citizen science is the collection or analysis of data by amateur or nonprofessional scientists, for example Evolution MegaLab, http://www.evolutionmegalab.org, Worthington et al. 2012). Within ecology, such apps have been used most widely to record the location of plants and animals, to date and georeference phenological events such as flowering, and to identify and locate invasive species. Apps are also used to implement crowdsourcing approaches to data handling and pattern identification (crowdsourcing is the subdivision of big or tedious jobs to a large number of people). For example, Zooniverse (http://www.zooniverse.org/) projects include seabed surveys where the users identify key features from underwater photographs, and species identification where users categorize recorded bat calls. Citizen science and crowdsourcing have been comprehensively reviewed elsewhere (e.g., Silvertown 2009; Conrad and Hilchey 2011; Gura 2013). Further, common uses of apps include public engagement and education, for example allowing automated dissemination of results in real time, or custom information based on location (this is well reviewed in Palumbo et al. 2012; Price et al. 2012). A good example is the customizable smartphone app designed by Price et al. (2012), which allows a geospatial approach to science education. An additional, perhaps less commonly utilized function is as a direct tool for data collection for individual researchers in the field, allowing easy contribution of data to a centralized database, and two-way communication between the researcher and the database. For example, EpiCollect allows GPS-tagged data collected by multiple field workers to be submitted to a smartphone and automatically displayed on a map, and other fieldworkers can then request and display this information on their own smartphones (Aanensen et al. 2009). Lwin and Murayama (2011) suggest that a similar system could be of particular use for gathering and sharing information such as meteorological data and damage information in disaster zones globally in real time. In addition to apps motivated by research, other potential sources of data exist as part of “citizen-initiated” databases and collection apps for diverse uses. Examples include forage.rs (http://forage.rs/) and boskoi (http://boskoi.org/), which are used for geotagging edible plants in urban locations and in the general landscape. Although not intended for research use, such resources may inadvertently provide open data of use to an ecological researcher. In recognition that this is a burgeoning field with new apps being released regularly, we have avoided an attempt to comprehensively list available apps; however, such lists are available online (e.g., http://brunalab.org/apps/) or by searching directly on a smartphone app store.

**Figure 1:**
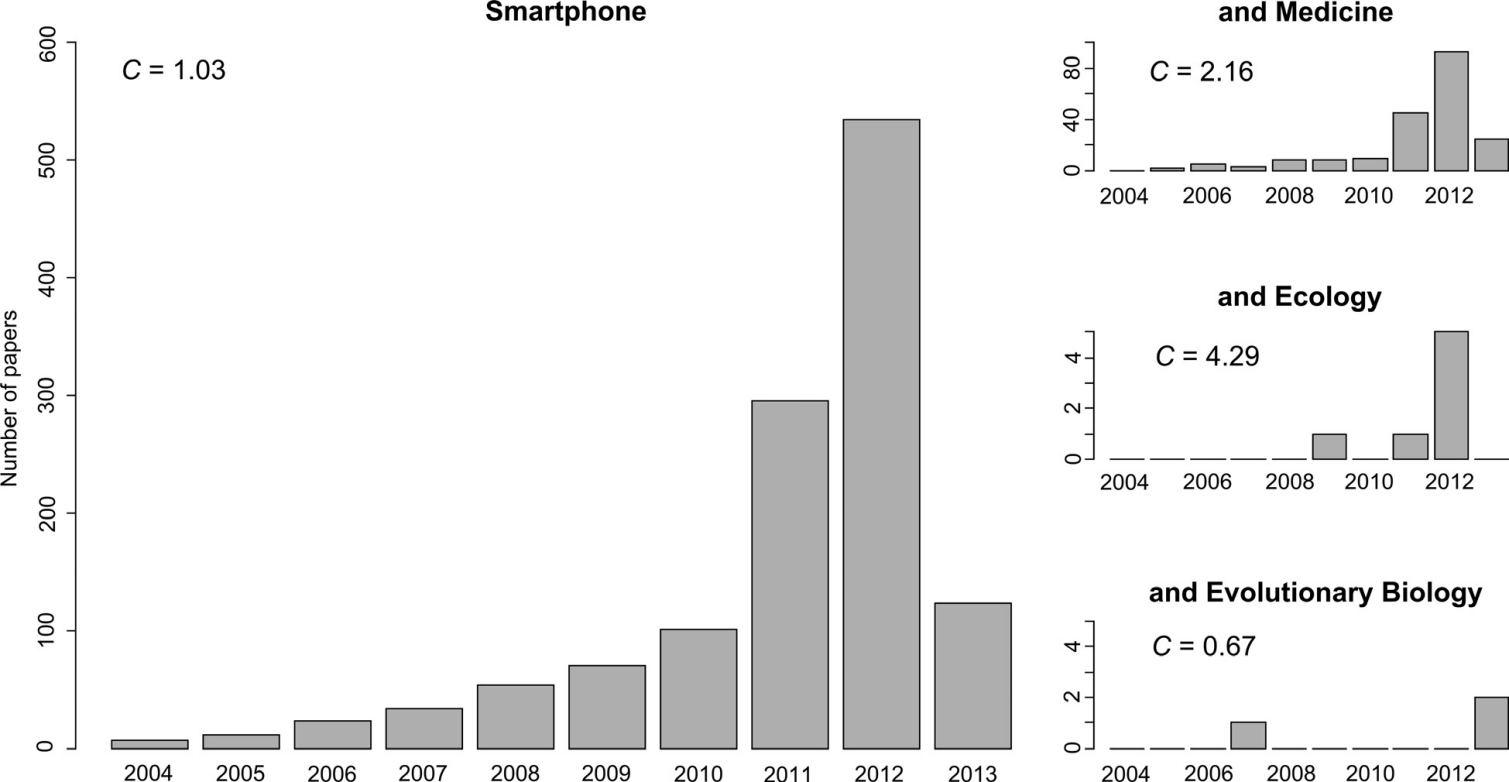
Results from bibliometric searches. Barplots show the number of papers published each year from 2004 to 2013, found using topic searches in Web of Knowledge to include the term “Smartphone.” Filtered searches shown in subplots compare the results of searches using {Smartphone AND Medicine}, {Smartphone AND Ecology}, or {Smartphone AND Evolution AND Bio*} (note that the “Bio*” wildcard was used since the term “Evolution” has several meanings and is commonly used in the software engineering literature). “*C*” describes the average number of citations to papers in each of the bibliometric searches.

Despite the large number of apps available, the literature on how these apps were developed and used is very limited, and as such there is currently a barrier for app-rehensive researchers to develop their own apps. At the time of writing, a search for the keyword “smartphone” in the Web of Knowledge database (Thomson Reuters, http://wokinfo.com/) revealed 1258 publications (starting in 2003 and increasing exponentially since; Fig. 1); however, many of these articles appear in engineering-related journals and conference proceedings. Repeating this search but filtering into the narrower scientific disciplines of “medicine,” “ecology” and “evolution” demonstrates that while there appears to be an app-idemic in medical research yielding 100 publications, ecological and evolutionary research has been slower on the uptake and publication of smartphones and apps. Ecology yielded seven publications, and evolutionary biology yielded only three publications (Fig. 1).

Here, we aim to plug the gap in the literature, addressing the main challenges of developing an app and outlining the development process. We focus primarily on apps for research (as apps for citizen science and education have been reviewed elsewhere, see earlier); however, much of the process will be similar when developing apps for education or other purposes. We describe two case studies of research-driven apps that we have developed, in order to highlight contrasting design issues and two very different development routes. Finally, we consider the future of smartphone apps in ecological and evolutionary research.

## The Development Process

**Figure 2:**
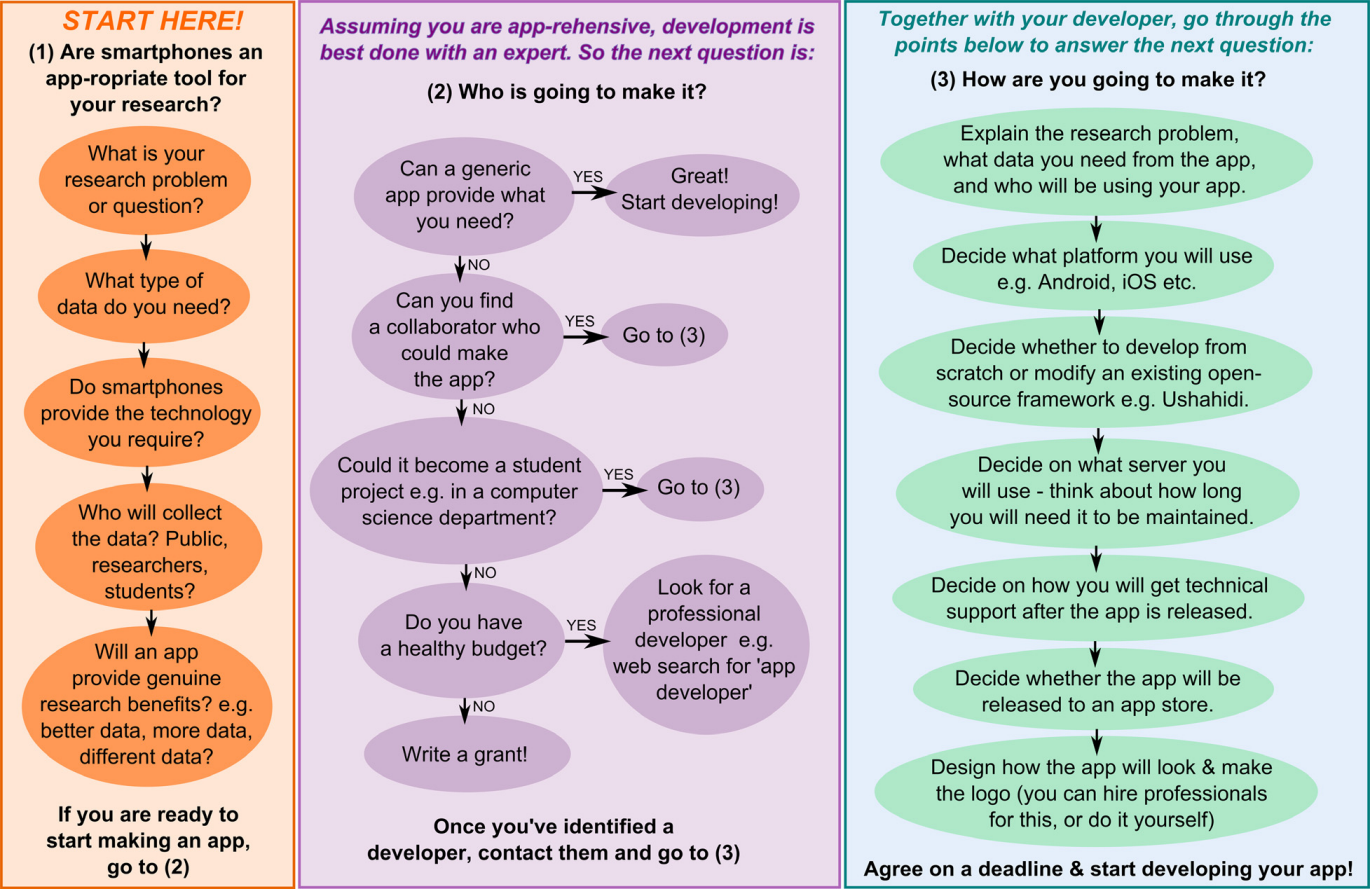
Outline of the development process.

Below, we describe three key stages of developing an app: Firstly deciding whether smartphones are an appropriate tool for your research, secondly deciding who will make your app, and thirdly outlining a number of key questions that you will face during the development. These are also summarized in Fig. 2. Clearly, each app will come with specific challenges that must be addressed, however we hope that this section will aid those interested in using an app for research to understand the broad development process from start to finish and to provide a basis for initiating a dialogue with developers.

**Figure 3:**
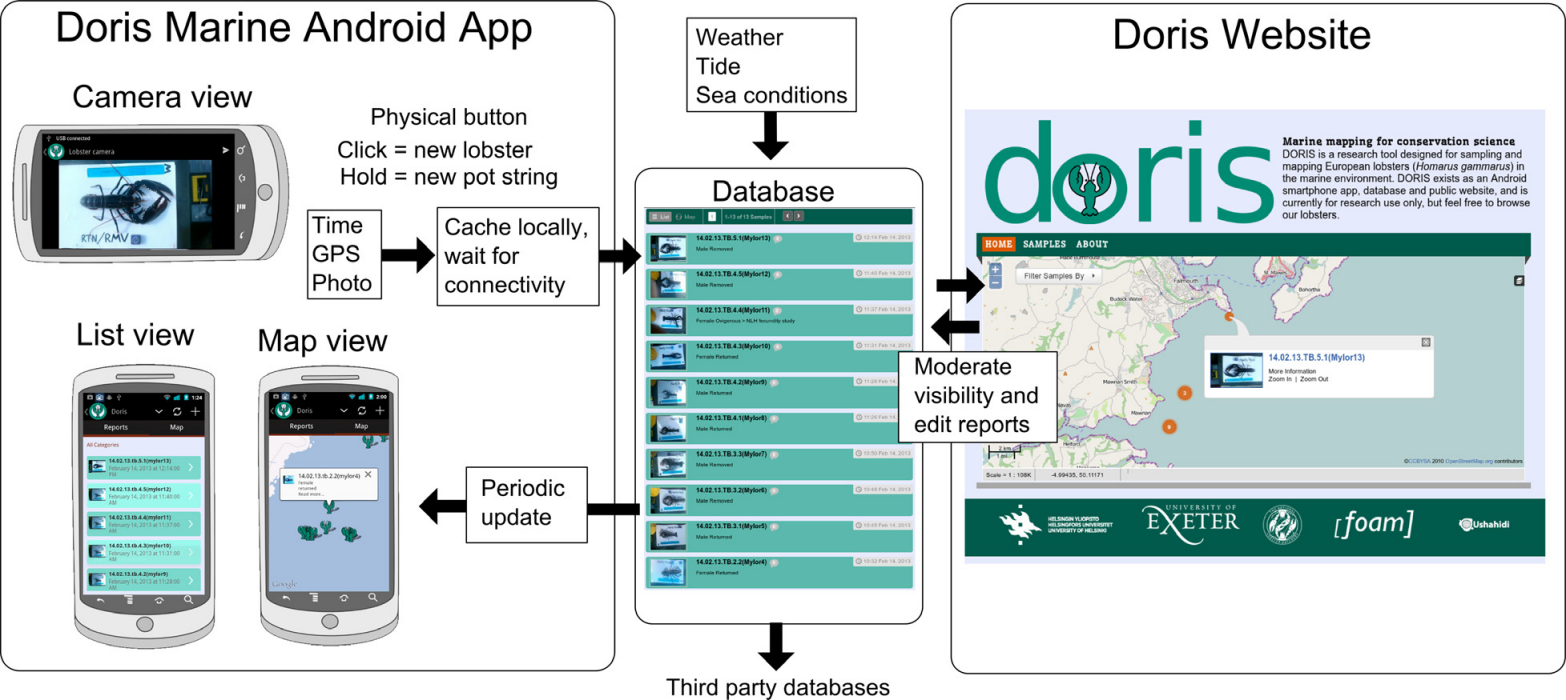
Schematic showing the interactions between the Doris Android App, database, and website.

### Are smartphones an app-ropriate tool for your research?

Before embarking on developing an app, the first steps are to determine a clear research question or problem to decide exactly what data are required and to establish whether this data can be collected using the technology and target audience available. Ideally, these questions should be thought through carefully before contacting a developer.

#### Identifying whether an app will be beneficial for your research

A clear challenge for the development of apps is to target specific questions where smartphone technology offers a benefit. The development of an app should ideally be driven first and foremost by a specific research problem, as opposed to being driven by the technology. Some potential benefits of apps could be:

- Wide geographical reach (e.g., you might attract users in many different countries to report on phenological events, avoiding the costs and time associated with fieldwork).
- The ability to collect data more efficiently (e.g., replacing old-fashioned data loggers that are not fit for purpose).
- The ability to collect data more accurately (e.g., avoiding human error by simultaneously recording location via GPS, the time, appending a photograph, and automatically uploading to an online database).
- Providing automatic backup while in the field (data can be stored on the smartphone SD card and uploaded to an online database).

#### Generating data of genuine use for research

Some examples of ecology-related apps to date make good use of smartphone technology in remarkably simple ways. For example, the Moose Survey app (University of Alberta) was designed for use by hunters in Alberta to send data on the number and location of moose seen (automatically logged using GPS, which does not require connectivity) to contribute to long-term population trend data. The data are stored on the SD card and then automatically uploaded once the user is within phone reception. Moose Survey requires the hunting licence number of the user, in order to verify the data. The Great Koala Count (Atlas of Living Australia) app takes a similar approach for gathering abundance and distribution data in Australia, but targets the public in a citizen science approach. These two examples have straightforward goals that are made transparent to the user, have very simple user interfaces, utilize the technology on the smartphone (in this case GPS, touchscreen, internal storage, and internet connectivity), and will generate research-quality data that would otherwise have been difficult and costly to obtain. Evaluating the available sensors on smartphones is an important step for deciding whether they might help you to collect usable data (such sensors are listed in the introduction).

#### Considering who will use the app

Apps developed for education or citizen science must be appealing to a wide range of users and must be engaging enough to ensure longevity of use. Those developed for citizen science projects face the key problem of balancing usability with the need to generate data that are genuinely useful for scientific research. In contrast, apps developed as data collection tools can focus more on generating suitable data and less on usability. These challenges can be addressed by understanding and keeping in mind the needs of the specific users throughout the planning and development process.

### Who is going to make your app?

The next issue to consider is who is going to make your app. There are many options, including developing the app yourself, collaborating with a researcher with the necessary expertise, developing a student project centered around making the app (perhaps in a computer science or a design department), collaborating with or hiring a freelance programmer, or outsourcing to a professional development company. The answer will largely depend on coding expertise, time, and financial constraints.

#### You

Self-development of certain types of apps has been facilitated by the recent development of a number of generic platforms targeted at natural science applications, such as Epicollect (Aanensen et al. 2009), iNaturalist (http://www.inaturalist.org), and Spotter (http://conserve.io). Both Epicollect and iNaturalist provide platforms enabling the user to set up specific projects to collect data (including georeferenced data and photographs) via the web or from smartphones and view the data centrally via a website. Spotter is a generic app and platform developed by the organization “Conserve.IO” which offers to work with researchers in the development of apps for specific research projects. In addition, similar results can be obtained using recently released field data collection apps such as Fulcrum & Fieldtrip GB (University of Edinburgh). These generic platforms can greatly simplify the app development process, particularly if fairly simple data collection is all that is required, and as such we recommend investigating these options first. On the negative side, the use of generic platforms will limit the extent to which the researcher can customize and add new feature to the apps. If the research question requires a customized app and the researcher does not have the skills or resources to develop the app themselves, there are a number of other options.

#### A collaborator or student

Many universities have departments housing accomplished programmers, who may be excited to work on an app as a collaborative effort. Furthermore, there are potential teaching opportunities as the development of an app could itself become a student project.

Additionally there are freelance programmers who may be willing to collaborate or work at reduced rates on a project that they find interesting of which brings them new and desirable skills. We recommend exploring these options before considering hiring a professional freelance developer or company to make your app.

#### A hired professional

There is a rapidly growing number of businesses specializing in custom app development, and they can easily be found using web searches. An app developed by a freelancer or a development company will likely cost between £3000 and £10,000 depending on its complexity, and the development time may be in the order of 1–3 months (note that these are broad estimations and may vary considerably, and may change over time). If open-source software is a requirement, professional development companies might not be suitable, as they may retain copyright over the product (though depending on the developer, it could also be possible to agree a contract whereby the researcher owns the code and graphics etc.). A good developer will implement user-centered design, perhaps accompanying you into the field to understand the unique challenges you face, or testing the app on a small group of public volunteers. A good developer will also use an “Agile” method of software development which involves incremental development, where the requirements and solutions are adaptive, for example, in order to deal with unforeseen issues that come up on testing the app or after release (Martin 2003).

### How are you going to make your app?

Here, we outline the biggest questions that you will face during the development of an app. Thinking about these in advance will enable you to enter into a productive dialogue with a developer; however, a developer should be able to guide even the most app-rehensive through these stages.

#### Choosing an operating system

One of the early decisions that must be made is which smartphone operating system to use. Currently, there are a number of different operating systems available for smartphones, including Google Android that is available on a wide range of smartphones, iOS for Apple devices, Microsoft Windows Mobile, and Blackberry OS, among others. By far, the most popular operating systems are Android and iOS. Android is the most widely used system found on the largest range of smartphones, and is the most amenable to making the app, and its associated code, open source. One disadvantage of Android is that as it runs on many smartphones with different specifications, ensuring that apps run smoothly on all phones can be problematic and potentially result in substantially greater development time. This is less of an issue for iOS as Apple has a much smaller range of devices, although Apple applies a much more stringent and restrictive process for submitting apps to the App store.

#### Modifying an existing system or starting from scratch

Platforms providing open-access source code for relatively generic smartphone apps, database schema, and websites are available, such as the Ushahidi platform (http://www.ushahidi.com), which was developed primarily for political monitoring applications (Okolloh 2009). Such platforms can be modified for custom uses by a skilled programmer (e.g. the DORIS app described in the case study below was created in this way), and as a side benefit, this approach contributes back to the open-source community. However, we recommend being cautious about this approach as although it could save considerable time by providing prewritten code, these platforms are not always coded efficiently and may provide headaches that could be avoided by writing entirely custom code.

#### Establishing a server, database, and website

In addition to developing the app itself, most apps will need server integration to allow the data collected to be centralized and accessed by the researcher. It may be possible to find a server located within your University Department, which is free to use. If a server is provided externally, for example by an app development company, you must be aware that the server may require a licence to be renewed regularly, otherwise the app will cease to function.

In addition, many projects will require an online database for storing data uploaded via the app and perhaps also a website where the data collected can be displayed or mapped. These can be made public or kept private for example through the use of a log-in system or a mixture of the two (e.g., a public map displaying some samples, and a private database where these samples can be moderated and edited). In cases where data are collected by the public, it will most likely require some moderation before it can be used for research purposes, and this is likely to take place via the online database. A number of mapping solutions (e.g., Google Earth, QGISCloud, and MapBox) facilitate the production of maps, which can be embedded within the project website and the app itself.

#### Peripheral issues

There are a number of peripheral issues that must also be considered, which may add to the time and financial requirements during the development phase. Most apps will require some graphics, at the very least an icon or logo to be displayed on the smartphone, which may in some cases require the skills of a graphic designer. The app will need to be added to Google Play App Store and/or to Apple App Store to allow public access and to allow updates to be automatically uploaded to users' smartphones. In addition, ongoing technical support may be required after release and will need to be agreed upon with the developer.

Below, we provide two case studies for ecological apps, which we have developed, outlining the background to the research problem, the development process, and the specific design issues that were addressed. Our aim is to showcase the starkly different development routes and end goals that can be pursued in order to produce an app of genuine use for ecological data collection and research. One case study, DORIS, provides an example of an app developed as a researcher tool, with a collaborative and open-access development process. The second case study, Magpie Mapper, provides a contrasting example of an app developed for citizen science, using a commercial app development company.

## Case Studies

### Case study 1 – DORIS (developed by AGFT, DJG, DJH)

#### Background

DORIS was designed to address a specific sampling issue for a project relating to European lobster (*Homarus gammarus*) population genetic structure (Fig. 2). Lobster sampling takes place on commercial fishing boats and so must be performed quickly and without interruption to those working on the boat. For each individual lobster, a unique ID code, geographical coordinates, time, date, and a photograph (for sizing and sexing) were required to accompany each genetic sample. A smartphone app was developed to avoid the need for a researcher to carry a separate camera, datalogger, and GPS device, as this was deemed to be impractical on a working boat and the time required to store these data for each individual manually was prohibitive. Automating the process via an app reduces human error and takes advantage of the fact that the user would always carry a smartphone as an essential piece of safety kit. The app was custom made as no generic apps provided all the requirements. The main functions required from the smartphone application were

1. Photograph each individual lobster placed next to a ruler for sizing.
2. Generate a unique ID number for each individual, encompassing (i) the sampling trip name/number, (ii) a number referring to the string of lobster pots within each sampling trip, and (iii) the individual lobster within each string of lobster pots (the user can then write this number on a tube for the genetic sample in order to match the sample with the record).
3. Georeference each individual.
4. Log the time and the date that each individual was sampled.
5. The potential to use the geographical coordinates and time/date information to link automatically to environmental datasets to provide weather and tide data for each sampling event.

Two key obstacles were identified; the first being that the system needed to be easy and quick to use while sampling at sea, potentially wearing gloves in a wet and unstable environment, ruling out the use of complex touchscreen interfaces. The second obstacle was that the system needed to function outside of mobile and/or wi-fi reception range, as internet connectivity could not be expected while at sea.

#### Application development and design features

The developer was a freelance software artist (DJG), who worked in close association with the researchers – going into the field in order to gain insights into, for example, the restrictions of working on small fisheries' boats. This employed a participatory design approach (Schuler and Namioka 1993), which describes ways to enhance understanding of the users, their motivations, and working environments and to use this information during the design process. DORIS was built on the free open-source Ushahidi platform (http://www.ushahidi.com) and is hosted on a server at the University of Exeter. The app was developed for Android, with a substantial rewrite of the Ushahidi android app, programmed in Java. The web application (written in PHP) required little modification beyond style changes and removing unnecessary features. Source code for DORIS is available under open-source GPL license and can be found on GitHub at http://github.com/nebogeo/doris. The use of, and contribution to, established open-source frameworks and standards allowed fast start-up, as changes could be made to existing code, and often solutions already existed for problems that were encountered. Involvement of existing interested open-source software communities resulted in added exposure, for example Ushahidi showed interest in DORIS and announced the project on their Twitter feed. Fast online updates provided by the Google Play Store allow rapid cycles of design based on continuous feedback from the users involved, a key tenet of Agile methodology (Martin 2003).

The majority of the modifications centered on improving stability of the smartphone application, simplifying the interface, and addressing internet connectivity issues so that the application could function offline. For DORIS to function, GPS signal must be receivable at all times, however, mobile reception and internet connectivity are assumed to be patchy. Our assumption of GPS signal being receivable is reasonable as the sampling will take place at sea – GPS should work anywhere on Earth with an unobstructed line of sight to four or more satellites and in any weather conditions.

As DORIS was designed for sampling at sea, the smartphone needed to be in a waterproof case and the user would be wearing chunky waterproof gloves, prohibiting the use of touchscreen buttons. Instead of using the touchscreen, a single physical button can be used, chosen to be one of the volume keys (which are present on most Android models). However, DORIS can function with the touchscreen as an alternative to physical buttons if required. All records are stored on the SD card, and then, once the smartphone enters an area with internet connectivity, the records are automatically uploaded to the web. By default, once the records have uploaded, they are removed from the SD card to make space. To protect the smartphone from the elements during marine sampling, a standard waterproof case from Overboard (http://www.over-board.co.uk) was used, together with a cord to tether the smartphone to the user. Additional information can be stored in the photograph through the use of physical indicators – a white plastic board is used for a base for photographing the lobster, and a ruler is set next to the lobster for scale. The ruler is moved toward an “F” or “M” on the board to indicate sex, and another physical marker (a colored block) is used to indicate whether the lobster is “berried” (laden with eggs).

Data from the DORIS smartphone application are sent to the DORIS database and are then viewable on a website (http://dorismap.exeter.ac.uk/). As DORIS is publicly available via the Android Marketplace, the administrator approval step protects against unwanted reports. The database can be directly accessed via the website, and SQL queries can be written to choose the raw data required (e.g., sample ID, geographical coordinates, date, time). This data can then be exported in various formats such as csv, Excel, or pdf files, making it ideal for direct use in research.

DORIS was produced in approximately 1 month in 2013, costing £3500 at a reduced freelancer rate due to the collaborative nature of the project.

### Case study 2 – Magpie Mapper (developed by RI)

#### Background

It is becoming increasingly apparent that human interactions with wildlife can have positive effects on physiological well-being (Fuller et al. 2007; Dallimer et al. 2012) although data on where and when these interactions take place are lacking. For many people, their most common encounters with wildlife are through birds. Indeed, birdwatching is the fastest growing sector of ecotourism (Ṣekercioḡlu 2002). There are numerous smartphone apps aimed at birdwatchers, including electronic field guides, but also apps to record sightings of different species of birds, including eBird (Wood et al. 2011), which was among the first citizen sciences projects to offer a smartphone application to record data. While apps such as these offer an exciting opportunity to collect a rich array of ecological data, they also require the user to be skilled in bird identification. In contrast, Magpie Mapper aimed to target the general public without specialized bird ID skills in order to investigate where and when people generally interact with wildlife. The app was required to be as easy to use as possible, simply recording sightings of a single species, the magpie (*Pica pica*). The magpie was used as a study species as it is highly distinctive and easy to identify, and is a common species found in a wide range of habitats. In addition, it is steeped in folklore and is a very divisive species, despised by some and loved by others, providing the necessary public appeal. The initial design for the app was simply that a user would spot a magpie and press a button within the app causing the device to record the GPS location, time, and predefined user ID. The data were then sent via the mobile phone network to a central server where it would be stored in a database from which the data could be remotely downloaded via a website. At the time of development, the generic platforms were either not available or unknown to the researchers; hence, the development of a custom app was considered to be the only option.

#### Application development and design features

The app was commissioned by the Environment and Sustainability Institute, University of Exeter, and development was carried out by App Future, Cornwall, UK. The researchers specified the app to have the following features:

1. User registration. On first use, users are asked to enter a valid email address along with details of age, sex, and level of education.
2. Home page. Most apps have a home page with brief instructions on how the app operates; indeed, this is a specific requirement for an app to be accepted on Apple's App store.
3. GPS recording screen. The main design feature was a large button with a magpie icon, which recorded the GPS location of the user once pressed. The button appears red until the phone obtains the GPS fix, and until this point does not allow data to be recorded. Once a fix has been obtained, the button turns green and a magpie sighting is recorded. Additional features include the ability to change the number of magpies recorded in any one sighting and to view the user's current latitude and longitude.
4. Log page. This displays all the magpie records recorded for that particular user, along with summary statistics for the whole project.
5. Further information page. This contains further details about the project, some basic magpie identification data, and links to the project website (but not the project database).

All app development, from production of graphics to page layout, app design, and submission to Apple App Store, were carried out by App Future in close collaboration with the researchers. App Future also developed integration of a computer server to store the data and production of the administration portal to access the data via the web. The app was initially designed and developed for iOS for the Apple iPhone and later converted for the Google Android operating system. The app is freely available for both operating systems. All data collected via the app is displayed using interactive maps powered by MapBox (http://mapbox.com/) on the project website (http://www.magpiemapper.co.uk) along with more details about the project. The total cost for the development of the apps for both iOS and Android, including costs associated with registering as a developer and server integration, was £4300. The development time, from initial concept to the app going live and collecting data, was 6 months, of which 5 months were collaborating with App Future on production of the actual app.

### Contrasting case studies

The case studies presented demonstrate two apps targeted at very different audiences and implementing divergent development processes. In both cases, app developers were engaged with the process at the earliest possible stage, however, DORIS was developed on a collaborative basis with a professional software artist (DJG), while Magpie Mapper was outsourced to a professional development company. In the case of DORIS, a collaborative approach was necessary due to the specialized nature of the conditions under which the app must function (marine fishing trips). Magpie Mapper was targeted at a public audience and so did not require field testing in the same way. While many of the features of each app are very similar (simple interface; GPS, time and date recorded; database and website integration), there were features that were unique to each app. DORIS required a single physical button to work the app, a photo to be uploaded, and had to work in areas with no mobile or wi-fi reception. In contrast, Magpie Mapper required a user login and instruction page as it was to be used by the public as opposed to individual researchers. The public target audience for Magpie Mapper also necessitated cross-platform development (iOS and Android), while DORIS was developed only for Android due to the benefits of the open-source approach and cheaper smartphones, which were adequate for the research-only purpose. The public audience for Magpie Mapper also necessitated more emphasis on design, and as such the design and graphics were outsourced, whereas DORIS graphics were produced at no cost by the researchers. Despite different user groups resulting in divergent design and development processes, the end results for Magpie Mapper and DORIS were remarkably similar; in both cases, simplification was key, allowing research data to be collected through a single click. For both case studies, it would not have been possible to obtain the required data through any other means other than an app, thus demonstrating the power of smartphones as research tools.

## Future Directions for Ecology Apps

As the ubiquity of smartphones increases both among the public and scientists, we expect their uses in citizen science and regular fieldwork to experience a matching increase. The adoption of smartphones in developing markets will lead to a change in the types of data available, and to the breadth of user groups. In 2011, Huawei in partnership with Safaricom released an Android phone costing $80 in Kenya, and in 2012, Google launched Free Zone allowing basic “feature” phones with internet connectivity to access Google products without the need for a smartphone. The announcement of Ubuntu Phone in early 2013 provides further opportunities for smartphones in the developing world. Ubuntu is a Linux-based operating system for PCs, and likewise, Ubuntu Phone will be free and open-source software, which runs on Android smartphoned. In addition, the Firefox OS phone, which launched in June 2013, will be targeted first at emerging markets. These types of advances will change the potential user groups for research-related apps over the coming years. One critical step for ensuring uptake of apps in developing countries is to optimize apps to reduce bandwidth use in order to keep the data costs down for the users. As more operating systems become available, choosing which to use (and thus which audience to target) will become more complicated. In addition, there will be fewer generic apps available for newer operating systems, and less existing expertise in these systems, and so development costs may be higher.

The technology associated with smartphones (also tablets and phablets) is also expected to continue to advance, to include, for example, better cameras, better microphones, increased storage capabilities/processing power, and a wider range of scientific sensors. As such, there is great potential for the use of this technology in research. Potential uses might include linking smartphones to external environmental sensors such as temperature or pressure sensors, using smartphones to record barcodes as tags on animals or using Bluetooth to collect data from GPS tags, and detecting individuals and/or species through direct computer vision or machine listening.

Smartphone apps could provide the image analysis required by high-throughput morphometrics and pattern analysis for the recognition of individuals (Bolger et al. 2012; Hiby et al. 2013), biodiversity, and habitat assessments from aerial photographs (Getzin et al. 2012), monitoring of microcosm populations (Pennekamp and Schtickzelle 2013); cell phenotype assays (Carpenter et al. 2006); the video tracking required in animal behavior research (Kross and Nelson 2011; Pennekamp and Schtickzelle 2013); and the extraction of biological signal from environmental sound recordings (Adams et al. 2012; Mennill et al. 2012). In cases like these where processing requirements are high, processor intensive algorithms can be run on a remote server, and the results transferred back to the phone. This approach is used for voice recognition by Apple's Siri application (http://www.apple.com/ios/siri/).

One of the clearest opportunities comes from the use of smartphones at the interface between ecological and social science applications. The natural link between the mobile phone and its owner makes it possible to collect detailed information on the owner's health, mood, and well-being. If coupled with environmental sensors and replicated across many (willing participant) smartphone users, this information could promote our attempts to understand how humans interact with, and benefit from, nature (Fuller et al. 2007).

A critical change for the power of smartphone apps for research will be the integration of data collected in the field or by citizen scientists with centralized databases, for example weather or pollution data. In addition, there is an exciting opportunity to develop a truly generic, open-access platform for apps for ecological or evolutionary research, allowing researchers to customize an existing system rather than employing developers each time a new application is required. Another area with potential to lower the costs of development is using web technology to build apps (HTML5). This greatly eases problems with making apps that work on different platforms, but at the cost of making offline use more complex, difficulty accessing sensors, and the use of more demanding algorithms (e.g., image processing), thus rendering this approach potentially prohibitive. However, in the near future, we expect this to become more realistic for rapid and cheaper development for some types of application.

As graphics and camera technology in smartphones develop and gradually move to different forms such as Google Glass, where information is overlaid on our view of the world, we see potential in the biological sciences for advances in Augmented reality. Such techniques allow scientific data to be presented by being linked directly with the world it derives from, rather than being restricted to abstract representations. This technique will provide new, rich methods for communication of scientific data to engage with broad audiences in the natural environment, as well as more flexible tools for scientists to visualize and interact with their models and data in the field.

It is an exciting time for the use of technology in ecological research: The phone in our pocket deserves better application as a collector and handler of data, while its inbuilt links to the internet reveal the potential for real-time information flow between collectors, databases, interpreters, and users of information. These technologies provide an unusual opportunity to link science with society, as well as to revolutionize the ways in which we collect our data.

## Data Accessibility

DORIS is available through Google Play App Store, and the website can be accessed at http://dorismap.exeter.ac.uk/. All source code is open source and available for free at http://github.com/nebogeo/doris. Magpie Mapper is available from the Apple App Store and Google Play App Store. The website can be found at http://www.magpiemapper.co.uk.
